# Clinical and Prognostic Value of VHL in Korean Patients with Rectal Cancer

**DOI:** 10.3390/medicina61020306

**Published:** 2025-02-10

**Authors:** Sang-Won Moon, Jun-Chae Lee, Jae-Ho Lee, Tae-Young Kim, Jong Ho Park

**Affiliations:** 1 Medical Course, School of Medicine, Keimyung University, Daegu 42601, Republic of Korea; swmoon94@naver.com (S.-W.M.); chai1703@naver.com (J.-C.L.); 2 Department of Anatomy, School of Medicine & Institute for Medical Science, Keimyung University, Daegu 42601, Republic of Korea; anato82@dsmc.or.kr

**Keywords:** VHL, TCGA, rectal cancer

## Abstract

*Background and Objectives*: Von Hippel–Lindau (VHL) disease is caused by mutations in the VHL gene and can develop various cancers. Hypoxia-inducible factors 1 and 2 alphas, regulated by the VHL gene, can increase the levels of vascular endothelial growth factor, thereby activating cancer progression. Here, we demonstrated clinical and prognostic values of VHL expression in rectal cancer (RC). *Materials and Methods*: Von Hippel–Lindau mRNA expression was examined in 60 patients with RC. Furthermore, we evaluated survival to determine the prognostic significance of VHL mRNA expression levels in RC using the Cancer Genome Atlas (TCGA) data. *Results*: Lower VHL expression was correlated with the recurrence (*p* = 0.058) and lymphatic invasion (*p* = 0.078), although it was not statistically significant. In TCGA data, VHL expression level was correlated with the M stage (*p* = 0.044); however, it had a possible association with lymphatic invasion (*p* = 0.068) and N stage (*p* = 0.104). Survival analysis showed that lower VHL gene expression predicted poorer survival in both patients with RC and TCGA data. *Conclusions*: This study identified a significant correlation between VHL gene expression and RC for the first time using patient tissues and TCGA data, suggesting that the VHL gene expression level could be a potential biomarker or candidate for the treatment of RC. Further studies are required to identify the molecular pathogenesis and clinical characteristics of VHL disease in RC.

## 1. Introduction

Colorectal cancer (CRC) ranks as the third most frequently diagnosed cancer and holds the third highest mortality rate among all cancers in South Korea. It is one of the most prevalent malignancies globally and the second leading cause of cancer-related deaths in the Western world [1,2]. The pathogenesis of CRC involves both genetic and environmental factors. The majority (70–80%) of CRCs are sporadic, whereas 20–30% of CRCs are associated with inherited factors, including Lynch syndrome (3–4%) and familial adenomatous polyposis (approximately 1%) [3]. The major pathogenesis of CRC is genetic instability, which can be advanced by at least two mechanisms [4]. The adenoma–carcinoma series primarily explains most human CRCs with the accumulation of mutations involving the APC, MMR, K-ras, DCC, and p53 genes [5]. Although the molecular genetics of CRC pathogenesis have been widely studied, further studies are needed.

Von Hippel–Lindau (VHL) disease is an inherited genetic disorder caused by somatic mutations in the VHL gene on chromosome 3 [6]. It can develop into pheochromocytomas, pancreatic neuroendocrine tumors, pheochromocytomas, retinal and central nervous system hemangioblastomas, clear cell renal cell carcinomas (RCC), and endolymphatic sac tumors [7,8]. The VHL gene encodes the pVHL protein, which functions as a tumor suppressor by acting as the substrate recognition component in the ubiquitin E3 ligase complex [9]. Two key proteins regulated by the VHL gene are hypoxia-inducible factor 1 (HIF1A) and 2 alpha (HIF2A) [10]. When VHL-induced degradation is impaired, HIF1A and HIF2A accumulate, resulting in elevated levels of erythropoietin (EPO), vascular endothelial growth factor (VEGF), and other growth factors, which promote tumor growth [11,12,13]. The VHL gene also plays a crucial role in maintaining primary cilia, regulating cytokinesis, controlling microtubule function, preserving extracellular matrix integrity, and regulating the cell cycle [14,15,16]. While several studies have explored the link between the VHL gene and various cancers, the connection between the VHL gene and colorectal cancer (CRC) remains largely unexplored.

The Cancer Genome Atlas (TCGA) is a research consortium that provides valuable data for investigating various genes involved in cancer [17,18]. Our preliminary study indicated that VHL expression may have prognostic significance in rectal cancer (RC). Thus, the objective of this study was to examine the clinical and prognostic implications of VHL gene expression in Korean patients with RC, as well as its correlation with the expression of other genes in colorectal cancer (CRC) using TCGA data. This study identified a significant correlation between VHL gene expression level and RC for the first time using patient tissues and TCGA data, suggesting that the VHL gene could be a potential biomarker or candidate for the treatment of RC.

## 2. Materials and Methods

### 2.1. Patients and Tissue Samples

A total of 60 patients (average age 63.6 ± 10.5 years, range 34–83 years) who underwent rectal cancer (RC) surgery at Dongsan Medical Center (Daegu, Republic of Korea) between April 2008 and January 2010 were included in the study. Tissue samples from both RC and adjacent non-cancerous tissues were obtained from the Keimyung Human Bioresource Bank, Republic of Korea. All patients were informed of the study‘s purpose, and informed consent was obtained from each participant. This study was approved by the Institutional Review Board of Keimyung University Dongsan Medical Center (No. 2020-07-027).

The clinicopathological data of each patient were reviewed. Patients with other malignancies were excluded. Additionally, patients with other malignancies and those who had received preoperative treatments, such as chemotherapy, radiofrequency ablation, or transarterial chemoembolization, were excluded from the study. The classification system of the American Joint Committee on Cancer 7th edition was assigned on the basis of the characteristics of the primary tumor (T) and the extent of regional lymph node involvement (N) and distant metastasis (M).

### 2.2. RNA Isolation and mRNA Expression Analysis

We extracted RNA from the tissues using the TRIzol reagent (Molecular Research Center Inc., Cincinnati, OH, USA) [19]. The RNA quality was measured using NanoDrop 1000 (Thermo Scientific, Wilmington, Denmark). Additionally, cDNA was synthesized from 2 μg of total RNA using M-MLV reverse transcriptase (Promega, Madison, WI, USA). Further, qPCR was performed using specific primers [20].

### 2.3. TCGA Data Analysis

**Figure 1:**
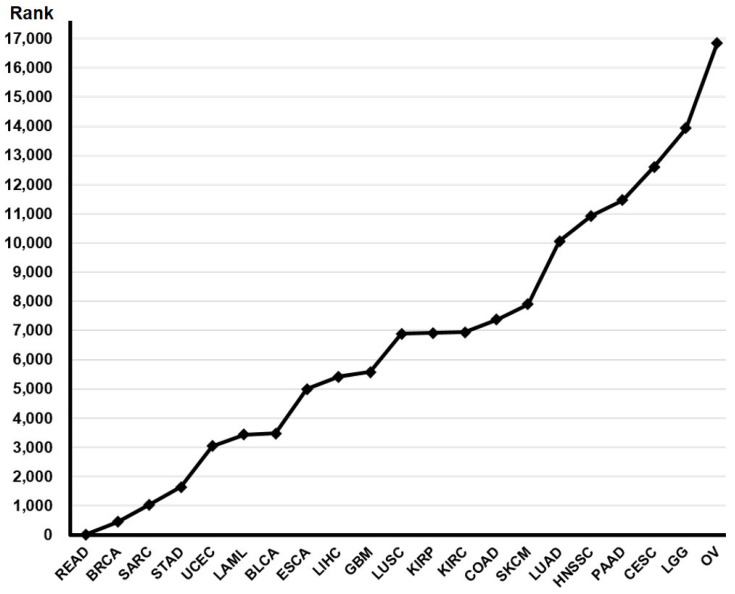
A *p*-value ranking for the prognostic value of VHL gene expression in various cancers. bladder urothelial carcinoma, BLCA; breast invasive carcinoma, BRCA; cervical squamous cell carcinoma, CESC; colon adenocarcinoma, COAD; esophageal carcinoma, ESCA; glioblastoma multiforme, GBM; head and neck squamous cell carcinoma, HNSC; kidney renal clear cell carcinoma, KIRC; kidney renal papillary cell carcinoma, KIRP; acute myeloid leukemia, LAML; brain lower grade glioma, LGG; liver hepatocellular carcinoma, LIGC; lung adenocarcinoma, LUAD; lung squamous cell carcinoma, LUSC; ovarian serous cystadenocarcinoma, OV; pancreatic adenocarcinoma, PAAD; rectum adenocarcinoma, READ; sarcoma, SARC; skin cutaneous melanoma, SKCM; stomach adenocarcinoma; uterine corpus endometrial carcinoma, UCEC.

We used primary data from the TCGA portal (http://cancergenome.nih.gov/, accessed on 1 February 2024) in February 2024. This provided a *p*-value ranking for the prognostic value of VHL gene expression in each cancer type. (Figure 1). The cancer type that demonstrated the most promising results, rectal cancer (RC), was selected for detailed analysis. A total of 158 RC patients were included in the clinical and survival analyses. Survival was defined as the time interval between surgery and death.

### 2.4. Statistical Analysis

Data were analyzed using SPSS software (version 25.0; IBM SPSS, Armonk, NY, USA). Clinicopathological characteristics, including age, sex, carcinoembryonic antigen level (CEA), and pathological TNM stage, were analyzed using the chi-square test. Spearman correlation was used for correlation analyses between the VHL gene expression and variables related to RC. The univariate survival analysis was performed using Kaplan–Meier curves and the log-rank test. Overall survival (OS) was defined as the time between diagnosis and mortality. Disease-free survival (DFS) was defined as the time between diagnosis and disease recurrence or development of distant metastasis. Statistical significance was set at *p* < 0.05.

## 3. Results

### 3.1. VHL Expression in Patients with Rectal Cancer

Von Hippel–Lindau gene expression was analyzed in 60 RC patients, then, divided into two groups according to their median values to identify the clinicopathological significance of VHL. Therefore, a higher VHL expression level was observed in 15 (25%) of 60 patients with RC. A high VHL expression level was associated with recurrence (*p* = 0.058); however, it was not significant statistically. Other clinicopathological variables were not associated with VHL expression (Table 1).

**Figure 2:**
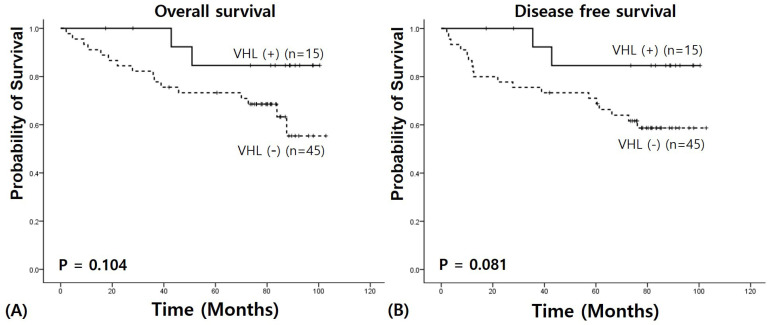
VHL expression survival analysis in rectal cancer: (**A**) Overall survival; and (**B**) Disease-free survival.

We performed survival analysis in RC to clarify the prognostic value of VHL mRNA expression. The median follow-up period was 81.96 months (range: 2–103 months). Survival analysis showed a longer OS in patients with RC with a higher VHL expression level (92.195 ± 5.376 vs. 77.592 ± 5.369 months, χ^2^ = 2.641, *p* = 0.104) (Figure 2). A higher VHL expression level had an association with longer DFS (91.010 ± 6.142 vs. 73.625 ± 5.814 months, χ^2^ = 3.046, *p* = 0.081).

### 3.2. VHL Expression in the Cancer Genome Atlas (TCGA) Data

The patients were divided into two subgroups according to the median values of VHL expression to show clinical characteristics of VHL expression in RC. The clinicopathological features of VHL mRNA expression in RC are presented in Table 2. A lower VHL expression was significantly correlated with metastasis (*p* = 0.044). Although not statistically significant, VHL was associated with lymphatic invasion (*p* = 0.068).

**Figure 3:**
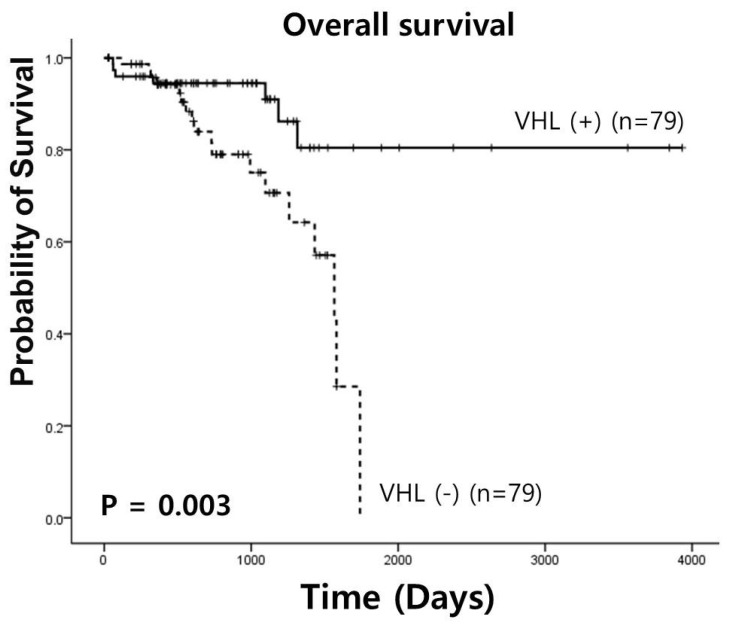
Univariate survival analysis in rectal cancer by TCGA database.

A univariate survival analysis was performed to determine the predictive value of VHL expression in RC (Figure 3). VHL expression had a statistically significant prognostic value in overall survival analysis (3341.68 ± 226.46 vs. 1326.62 ± 77.30 days, χ^2^ = 8.75, *p* = 0.003).

The correlation analysis between the expressions of VHL and other genes, such as APC, KRAS, and P53, was performed (Table 3). Continuous variables, such as age and carcinoembryonic antigen level, were also included. The correlation analysis demonstrated that APC expression was positively correlated with VHL (R = 0.206; *p* = 0.010) and KRAS expressions (R = 0.261; *p* = 0.001), and it was negatively correlated with age (R = −0.222; *p* = 0.005). The other correlations were not statistically significant.

## 4. Discussion

We examined the clinicopathological and prognostic potential of VHL expression in RC by patients‘ tissues and TCGA data. The VHL gene encodes a tumor suppressor protein that regulates the degradation of hypoxia-inducible factors (HIFs) involved in the cellular response to oxygen levels [6,8]. When the VHL gene is functional, its expression prevents excessive angiogenesis, thereby inhibiting tumor growth. Furthermore, VHL and tumors are closely related to clinical outcomes and their genetic information may predict patient responses before cancer treatment [21,22,23]. The loss of VHL gene expression is commonly associated with CRC progression and poor prognosis [24,25].

A lower VHL expression level was significantly associated with metastasis and lymphatic invasion. In tissues of patients with RC, a lower VHL expression level was associated with recurrence and the N stage. This result is in agreement with TCGA data. In addition, the VHL expression level is lower in patients with RC and lymphatic invasion. Although the difference was not statistically significant, a lower VHL expression level tended to be associated with the N stage. The loss of VHL expression can contribute to increased lymphatic invasion as an indicator of tumor spread through the lymphatic system, thereby worsening the overall clinical outcome of cancer.

Our survival analysis showed that a lower VHL expression level predicted poorer OS and DFS, which corroborated the TCGA data. A low VHL mRNA expression level is generally associated with poor prognosis and shorter survival in several cancers, particularly renal cell carcinoma (RCC) [26]. The loss or reduced expression of the VHL gene is common and is directly correlated with increased tumor size, lymph node involvement, and metastasis in RCC. This is consistent with our results regarding the association between the VHL expression level and lymph node metastasis. The loss of VHL function can lead to increased HIF stabilization and upregulation of pro-tumorigenic factors, such as VEGF, thereby promoting tumor growth and metastasis [10,11,12]. In particular, the clinical significance of hypoxia is linked to the activation of HIF, metastasis, resistance to chemotherapy and radiotherapy, and poor survival outcomes. This suggests that hypoxia may contribute to tumor progression and resistance to treatment [22,23]. The loss of VHL gene expression, which can occur through mutations, deletions, or silencing, is a hallmark of certain cancers and can lead to more aggressive tumor behavior and a worse prognosis [11,27]. These studies showed that VHL mutation at a particular site within exon 2 caused DNA damage, chromosomal instability, and other molecular statuses, leading to a poor prognosis [27,28]. Our correlation analysis showed a positive correlation between VHL and APC mRNA expressions. The loss of APC due to its mutation deregulates oncogene transcription, such as c-myc and cyclin D1, promoting poorer prognosis [29]. Although the correlation coefficient is not large, this suggests that VHL expression may be related to APC function. Therefore, VHL mRNA expressions in patients with APC and VHL mutations should be comprehensively studied.

This study has some limitations. We presented only univariate analysis without multivariate survival analysis because the large dataset in this study showed borderline statistical significance in the univariate analysis. Sixty patients with RCC were included, all of whom were men. Although the follow-up period was relatively long, the sample size was small. Additionally, the TCGA data for RCC had a small sample size (*n* = 158) compared to other cancers; therefore, large-scale studies with multivariate survival analysis are needed. Additionally, this study focused on RC based on survival results from TCGA data; however, research on colon cancer should be conducted. In some cancers, differences in RNA and protein expression levels showed inconsistent results [30,31]. Although our study showed consistent results in both patient tissues and big data, it is essential to demonstrate the correlation between RNA and protein expression levels to obtain clinical significance. Thus, additional experiments about protein expression and molecular mechanisms of VHL in colorectal cancer need to be elucidated.

## 5. Conclusions

This study identified a significant correlation between VHL gene expression and RC for the first time using patient tissues and TCGA data. This suggests that VHL gene expression level could be a potential biomarker or candidate for the treatment of RC. Overall, we emphasize the need for further studies, particularly larger case studies, on the molecular mechanisms and clinical characteristics of VHL in RC. The VHL gene may play a role in colorectal cancer progression and clinical features, paving the way for future research.

## Figures and Tables

**Table 1:** Clinical characteristics of VHL gene in rectal cancer.

	VHL		
	High	Low	*p*-Value
Age			
<65	7 (28.0)	18 (72.0)	0.650
≥65	8 (22.9)	27 (77.1)	
Recurrence			
No	14 (56.0)	31 (44.0)	0.058
Yes	1 (6.6)	14 (93.4)	
Lymphatic invasion			
No	8 (30.8)	18 (69.2)	0.367
Yes	7 (20.6)	27 (79.4)	
CEA			
≤5	12 (25.5)	35 (74.5)	0.856
>5	3 (23.1)	10 (76.9)	
Perineural Invasion			
No	11 (28.2)	28 (71.8)	0.435
Yes	4 (19.0)	17 (81.0)	
Differentiation			
Well	0 (0.0)	3 (100.0)	0.267
Moderate	15 (28.3)	38 (71.7)	
Poor	0 (0.0)	4 (100.0)	
M stage			
M0	13 (23.2)	43 (76.8)	0.232
M1	2 (50.0)	2 (50.0)	
N stage			
N0	9 (27.3)	24 (72.7)	0.078
N1	6 (37.5)	10 (62.5)	
N2	0 (0.0)	11 (100.0)	
T stage			
T1	2 (66.7)	1 (33.3)	0.116
T2	3 (37.5)	5 (62.5)	
T3	6 (15.8)	32 (84.2)	
T4	4 (36.4)	7 (63.6)	

carcinoembryonic antigen level, CEA.

**Table 2:** Clinical characteristics of VHL gene in rectal cancer by TCGA data.

	VHL		
	High	Low	*p*-Value
Age			
<60	24 (50.0)	24 (50.0)	0.958
≥60	54 (54.0)	55 (55.0)	
Gender			
Female	35 (49.2)	36 (50.8)	0.930
Male	43 (50.0)	43 (50.0)	
Lymphatic invasion			
No	46 (56.0)	36 (44.0)	0.068
Yes	23 (40.3)	34 (59.7)	
CEA			
≤5	30 (48.3)	32 (51.7)	0.870
>5	22 (50.0)	22 (50.0)	
Venous invasion			
No	52 (50.9)	50 (49.1)	0.591
Yes	16 (64.0)	19 (36.0)	
Pathologic stage			
Stage I	15 (50.0)	15 (50.0)	0.105
Stage II	29 (63.0)	17 (39.0)	
Stage III	22 (47.8)	26 (52.8)	
Stage IV	8 (33.3)	16 (62.7)	
M stage			
M0	63 (53.3)	55 (46.7)	0.044
M1	7 (30.4)	16 (69.6)	
N stage			
N0	46 (58.2)	33 (41.8)	0.104
N1	17 (39.5)	26 (60.5)	
N2	14 (43.7)	18 (56.3)	
T stage			
T1	5 (55.5)	4 (45.5)	0.294
T2	13 (46.4)	15 (53.6)	
T3	57 (53.2)	50 (46.8)	
T4	3 (25.0)	9 (75.0)	

carcinoembryonic antigen level, CEA.

**Table 3:** Correlation analysis in rectal cancer.

		VHL	APC	KRAS	P53	Age	CEA
VHL	R	1	0.206	0.037	−0.049	−0.025	−0.103
	*p*		0.010	0.646	0.540	0.756	0.294
APC	R	0.206	1	0.261	−0.148	−0.222	−0.013
	*p*	0.010		0.001	0.064	0.005	0.893
KRAS	R	0.037	0.261	1	−0.085	0.014	−0.007
	*p*	0.646	0.001		0.290	0.857	0.942
P53	R	−0.049	−0.148	−0.085	1	0.078	−0.104
	*p*	0.540	0.064	0.290		0.330	0.290
Age	R	−0.025	−0.222	0.014	0.078	1	0.008
	*p*	0.756	0.005	0.857	0.330		0.932
CEA	R	−0.103	−0.013	−0.007	−0.104	0.008	1
	*p*	0.294	0.893	0.942	0.290	0.932	

carcinoembryonic antigen level, CEA.

## Data Availability

The data presented in this study are available on request from the corresponding author.

## Abbreviations

The following abbreviations are used in this manuscript:

VHL	Von Hippel–Lindau
RC	Rectal cancer
TCGA	The Cancer Genome Atlas
CRC	Colorectal cancer
RCC	Renal cell carcinomas
HIF1A	Hypoxia-inducible factor 1
HIF2A	Hypoxia-inducible factor 2
EPO	Erythropoietin
VEGF	Vascular endothelial growth factor
OS	Overall survival
DFS	Disease-free survival
CEA	carcinoembryonic antigen level.
